# A New Duplex Recombinase Polymerase Amplification (D-RPA) Method for the Simultaneous and Rapid Detection of *Shigella* and *Bacillus cereus* in Food

**DOI:** 10.3390/foods12091889

**Published:** 2023-05-04

**Authors:** Shuna Xiang, Hanyue Zhang, Xiaoyan Cha, Yingting Lin, Ying Shang

**Affiliations:** Faculty of Food Science and Engineering, Kunming University of Science and Technology, Yunnan 650500, China

**Keywords:** recombinase polymerase amplification, duplex detection, *Shigella*, *Bacillus cereus*

## Abstract

*Shigella* and *Bacillus cereus* are two common foodborne pathogens that cause intestinal diseases and seriously affect human life and health. Traditional microbiological culture methods are time-consuming and laborious, and polymerase chain reaction (PCR)-based methods rely on expensive thermal cyclers and lengthy reaction times. In this study, on the basis of the specific gene *ipaH7* of *Shigella* and the virulence gene *nheABC* of *B. cereus*, a duplex detection system was established for the first time by using the recombinase polymerase amplification technique (D-RPA). After optimization, D-RPA could be effectively amplified at 42 °C for 25 min with excellent specificity, and the detection limits of D-RPA for *Shigella* and *B. cereus* in artificially contaminated samples were 2.7 × 10^1^ and 5.2 × 10^2^ CFU/mL, respectively. This study provides a certain research basis for multiple detection with RPA, an isothermal amplification technology. Furthermore, it lays a good foundation for high-throughput rapid detection of foodborne pathogens.

## 1. Introduction

*Shigella*, a Gram-negative bacillus, is one of the common pathogens causing intestinal diseases in humans; it is mainly found in dairy products, meat products, vegetables, and fruits [1]. *Shigella* is extremely pathogenic, with 10–100 CFU causing disease in adults [2]. The annual global incidence of *Shigella* is estimated to be as high as 165 million, of which 163 million cases occur in developing countries with most being fatal [3]. *Bacillus cereus*, a Gram-positive bacterium, is a common conditionally pathogenic bacterium that carries virulence genes that are important for its pathogenicity [4]. Statistics show that *B. cereus* accounts for 1.4–12% of global foodborne disease outbreaks [5], and the actual occurrence of food poisoning is far higher than this number. This pathogenic bacterium has been detected in almost all types of food and can contaminate a wide range of food, such as meat, dairy products, cereals, and condiments [6,7,8], causing vomiting, diarrhea, and many other types of illnesses [9].

Foodborne pathogenic bacteria are widely present in food and pose a great risk to human health. Therefore, the development of accurate detection methods for foodborne pathogenic bacteria is necessary and important for food safety and clinical diagnosis. At present, most methods used by food quality inspection departments and food enterprises for the internal detection of foodborne pathogenic bacteria are based on the traditional culture method, which takes 5–7 days to yield results and requires complex, cumbersome steps, such as culture, isolation, purification, and biochemical and serological identification [10]. With the development of various technologies, molecular biology techniques have become widely used in species identification studies due to their rapidity, specificity, sensitivity, and accuracy [11]. These techniques are mainly divided into the polymerase chain reaction (PCR)-based variable temperature amplification of nucleic acids and isothermal amplification techniques [12]. However, the earliest developed PCR techniques and their extensions rely on expensive thermal cyclers and complex detection steps to obtain their final results [13] and are thus unsuitable for primary laboratories or field testing. By contrast, the isothermal amplification of nucleic acids is particularly suitable for poorly equipped laboratories and field testing because of its sensitivity, rapidity, and low equipment requirements [14].

Recombinase polymerase amplification (RPA) is an isothermal rapid amplification technique, which mainly relies on recombinase, DNA polymerase, and single-stranded DNA binding protein (SSB). The reaction temperature is 37–42 °C, which can also be carried out at room temperature and reach the detection level within 20 min [15,16]. RPA has great advantages for detection in resource-poor laboratories and outdoor detection without complex instruments because of its high sensitivity and rapidity [17]. At present, the single RPA technique has made great progress and is widely used for the detection of foodborne pathogenic bacteria such as *Salmonella* [17], *Vibrio vulnificus* [18], *Staphylococcus aureus* [19] and *Listeria monocytogenes* [20]. Although the application of the multiplex RPA technique in the detection of foodborne pathogenic bacteria has been successfully reported [21,22,23], only a few studies on the multiplex RPA technique exist because it has a complex multiplex reaction system, its amplification efficiency is affected by many factors [24], and it easily produces false-positive results. At present, many of the enzymes involved in RPA are expensive, and the detection target is single. Therefore, the economy and high throughput will be the future development direction of RPA [25].

In this study, *Shigella* and *B. cereus* were used as the detection objects, and the multiplex RPA technique was explored. For the first time, a duplex detection system was established by using the RPA technique combined with electrophoresis (D-RPA), thus providing a method for the rapid and simultaneous detection of *Shigella* and *B. cereus*. This method is faster, more sensitive and convenient, and simpler to operate than traditional microbiology or PCR-based methods. Therefore, it has great application prospects and potential for the high-throughput rapid detection of foodborne pathogens. At the same time, this study provides a certain research basis for multiple detection by RPA, which is an isothermal amplification technology.

## 2. Materials and Methods

### 2.1. Materials

A total of 12 common foodborne pathogen strains, including 3 *Shigella* strains, 3 *Bacillus cereus* strains, and 6 other foodborne strains, were used to determine the specificity of the D-RPA assay in the study (Table 1). All strains were inoculated into a nutrient broth medium (Microbial Reagent Co., Ltd., Hangzhou, China) and cultured at 37 °C for 24 h. Bacterial concentration was determined by plate colony counting. All primers (Supplementary Materials Table S1) were synthesized by Sangon (Shanghai, China). All chemical reagents were of analytical grade.

### 2.2. DNA Extraction

DNA was extracted by using a DNA extraction kit (Ezup Column Bacteria Genomic DNA Purification Kit, B518255-0100, Sangon, Shanghai, China) in accordance with the instructions. DNA concentration and purity were measured by using a NanoDrop2000 microspectrophotometer (Thermo Fisher Scientific, Waltham, MA, USA), and DNA was analyzed on 1% agarose gel electrophoresis (AGE) containing TS-GelRed nucleic acid dye (0.1 µL/mL; TSJ002, TsingKe, Beijing, China). The extracted DNA was stored at −20 °C.

The extracted genomic DNA was subjected to PCR amplification by using 16S rDNA universal primers [26] to verify that it qualified for the subsequent experiments. The reaction system had a total volume of 25 µL and comprised 2.5 µL of 10× buffer, 2 µL of the dNTP mixture, 1 µL of each primer (10 µM), 0.2 µL of rTaq DNA polymerase (TaKaRa Biotechnology Co., Ltd., Dalian, China), 2 µL of template DNA, and 16.3 µL of ddH_2_O. The amplification procedure was as follows: initial denaturation at 95 °C for 5 min; 30 cycles of denaturation at 95 °C for 30 s, primer annealing at 58 °C for 30 s, and primer extension at 72 °C for 30 s; and final extension at 72 °C for 10 min. All amplification products were analyzed on 2% AGE.

### 2.3. Primer Design and Screening

The specific gene *ipaH* of *Shigella* and the virulence gene *nheABC* of *B. cereus* were obtained by referring to the literature [4,27,28]. The NCBI GeneBank (http://www.ncbi.nlm.nih.gov/BLAST) was searched for BLAST alignments. The *ipaH7* gene sequence (accession number: AE005674.2) and *nheABC* gene sequence (accession number: Y19005.2) with low homology were selected. In accordance with the design principle of TwistDX, primers were designed by using Primer Premier 5.0 software (Premier Biosoft, San Francisco, CA, USA), and BLAST comparative analysis was carried out to verify the specificity of the primers. All designed RPA candidate primers were diluted to 10 μM.

The primers that were most suitable for target amplification were then determined through PCR and RPA amplification. A 50 μL single RPA reaction system was prepared for determination in accordance with the TwistAmp^TM^ Basic kit (TwistDx, Cambridge, UK). First, the premix was prepared in a centrifuge tube; added with 2.4 μL of each primer, 29.5 μL of primer-free rehydration buffer, 2 μL of template DNA, and 11.2 μL of ddH_2_O for a total volume of 47.5 µL; and briefly, vortex shaken. The premix was added to lyophilized enzyme powder and mixed by pumping with a pipette gun. Finally, 2.5 μL of MgOAc (280 mM) was dropped onto the tube cover and centrifuged instantaneously to start the reaction. The reaction tubes were placed in an ABI SimpliAmp Thermal Cycler (Applied Biosystems, USA) and incubated at 39 °C for 20 min. The reaction tube was taken out, added with 25 µL each of the DNA extraction phenol reagent (Solarbio, Beijing, China) and trichloromethane (Chuandong Chemical Co., Ltd., Chongqing, China), and shaken through mixing after centrifugation for several minutes until stratified. The 5 μL supernatant was used for 2% AGE analysis.

### 2.4. D-RPA Reaction

The D-RPA detection system for *Shigella* and *B. cereus* was established by primer screening and verification. A total of 50 μL of the D-RPA reaction system was prepared in accordance with the TwistAmp^TM^ Basic kit Quick Guide. The reaction system was made up to 47.5 µL by adding 2.4 µL each of *Shigella* and *B. cereus* primers, 29.5 µL of primer-free rehydration buffer, 1 µL each of *Shigella* and *B. cereus* DNA as the template, 2.5 µL of MgOAc (280 mM), and ddH_2_O. The specific operation method is the same as that described in Section 2.3.

### 2.5. Condition Optimization

The key parameters, including primer concentration, reaction time, reaction temperature, and Mg^2+^ concentration, in the D-RPA detection system were optimized to obtain the best detection system. Experiments were set up with different primer concentrations at 30–45 °C, incubation times of 10–35 min, and the MgOAc addition gradient of 1–3.5 μL to determine the optimal reaction conditions. After RPA amplification, the results were examined through AGE, the grayscale of the electrophoretic bands was analyzed by using ImageJ software (NIH, Bethesda, MD, USA), and the peak area values reflecting the brightness of the bands were used to assess the feasibility of the optimized system.

### 2.6. Specificity and Sensitivity Analysis

In this study, three *Shigella* strains and three *B. cereus* strains were used to verify the specificity, and then six non-target strains (*V. parahaemolyticus*, *Salmonella*, *E. coli* O157:H7, *E. sakazakii*, *L. monocytogenes*, and *S. aureus*) and a mixture of target standard bacteria were used to evaluate the D-RPA reaction system.

For the evaluation of the sensitivity of the method, *Shigella* and *B. cereus* DNA were simultaneously diluted in a 10-fold gradient (30, 3, 0.3, 0.03, 0.003, and 0.0003 ng/μL), and the diluted concentrations of DNA were used as the templates for RPA amplification under the optimized conditions. The amplified products were detected on AGE.

### 2.7. Application of D-RPA in Food Samples

Frozen green beans were purchased from local supermarkets, and the samples were verified to be free of *Shigella* and *B. cereus* by using conventional PCR [29,30]. The samples were pretreated on a sterile bench by adding 2.7 × 10^1^–2.7 × 10^7^ CFU/mL of *Shigella* and 5.2 × 10^1^ –5.2 × 10^7^ CFU/mL of *B. cereus* to 25 g of samples, and placed in a sterile homogenization cup containing 225 mL 0.9% saline, homogenized at 8000–10,000 r/min for 1–2 min. DNA was extracted from 1 mL of artificially contaminated liquid sample, detected using the established D-RPA system, and compared with conventional PCR methods.

## 3. Results

### 3.1. Primer Screening for Shigella and B. cereus

**Figure 1:**
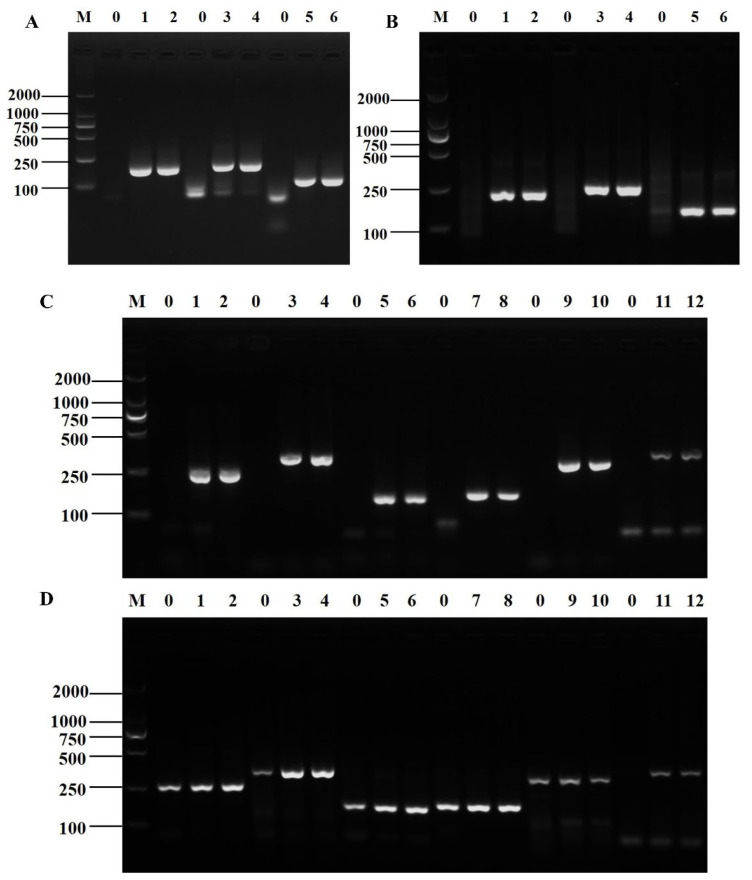
The verification results of candidate primers of *Shigella* and *B. cereus*. PCR (**A**) and RPA (**B**) amplification results of candidate primers of *Shigella*, Lane M: DNA marker DL2000; Lane 0: negative control; Lanes 1–2: *ipaH7*-1F/R; Lanes 3–4: *ipaH7*-2F/R; Lanes 5–6: *ipaH7*-3F/R. PCR (**C**) and RPA (**D**) amplification results of candidate primers of *B. cereus*, Lane M: DNA marker DL2000; Lane 0: negative control; Lanes 1–2: *nheA*-1F/R; Lanes 3–4: *nheA*-2F/R; Lanes 5–6: *nheA*-3F/R; Lanes 7–8: *nheB*-1F/R; Lanes 9–10: *nheB*-2F/R; Lanes 11–12: *nheB*-3F/R.

Three pairs of candidate primers for *Shigella* and six for *B. cereus* were validated through PCR and RPA amplification. The PCR amplification results of all three primer pairs of *Shigella* showed the expected length of the target band, and the electrophoretic bands were intact with clear brightness (Figure 1A). The RPA amplification products of the three pairs of primers were of the expected length with neat bands and clear and uniform brightness (Figure 1B). The electrophoresis results showed that all three primer pairs could specifically amplify *Shigella* genomic DNA. In combination with the established D-RPA reaction system, *ipaH7*-3F/R was finally selected as the specific primer for the RPA detection system for *Shigella*.

The PCR amplification results of the six primer pairs of *B. cereus* showed that the product size was as expected, and the electrophoretic bands were complete and bright (Figure 1C). The RPA amplification products of the six pairs of primers were as expected with neat and bright bands. However, the RPA amplification results of the first five pairs of primers had false positives, and only the sixth pair of primers had normal amplification results (Figure 1D, lanes 11 and 12). Therefore, *nheB*-3F/R was selected as the specific primer for *B. cereus* in the D-RPA amplification system.

### 3.2. Establishment of D-RPA Reaction System for Shigella and B. cereus

**Figure 2:**
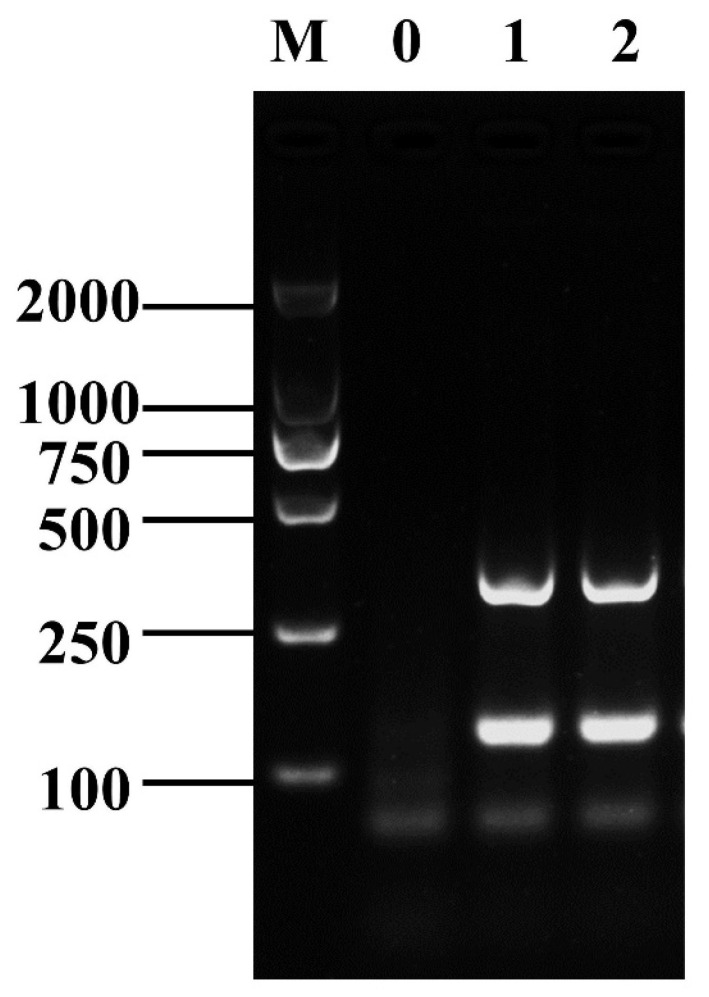
The establishment of the D-RPA reaction system. Lane M: DNA marker DL2000; Lane 0: negative control; Lanes 1–2: *ipaH7*-3F/R+*nheB*-3F/R.

The D-RPA reaction system was established by using the screened specific RPA primers of *Shigella* and *B. cereus*. The electrophoresis plots of the amplification results revealed that the amplification products of *ipaH7*-3F/R and *nheB*-3F/R showed small differences in band brightness, indicating low interaction, and the positions of the bands of both products were easy to observe and distinguish in the electrophoresis plots (Figure 2).

### 3.3. Optimization of D-RPA Reaction Conditions

**Figure 3:**
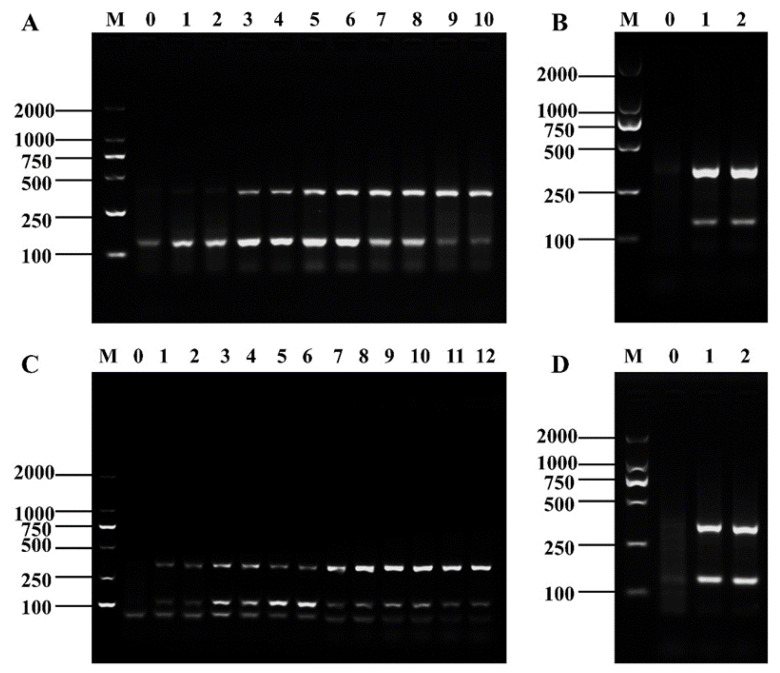
Primer concentration optimization results of the D-RPA reaction system. (**A**) RPA amplification results of same concentration combination of *shigella* and *B. cereus* primers, Lane M: DNA marker DL2000; Lane 0: negative control; Lanes 1–2: *Shigella*/*B. cereus* primer: 1.2 μL; Lanes 3–4: *Shigella*/*B. cereus* primer: 1.6 μL; Lanes 5–6: *Shigella*/*B. cereus* primer: 2 μL; Lanes 7–8: *Shigella*/*B. cereus* primer: 2.4 μL; Lanes 9–10: *Shigella*/*B. cereus* primer: 2.8 μL. (**B**) RPA amplification results of optimal concentration combination in (**A**), Lane M: DNA marker DL2000; Lane 0: negative control; Lanes 1–2: *Shigella* primer 2 μL + *B. cereus* primer 2.4 μL. (**C**) RPA amplification results of different concentration combinations of *shigella* and *B. cereus* primers, Lane M: DNA marker DL2000; Lane 0: negative control; Lanes 1–2: *Shigella* primer 2 μL + *B. cereus* primer 1.2 μL; Lanes 3–4: *Shigella* primer 2 μL + *B. cereus* primer 1.6 μL; Lanes 5–6: *Shigella* primer 2 μL + *B. cereus* primer 2 μL; Lanes 7–8: *Shigella* primer 2.4 μL + *B. cereus* primer 2.4 μL; Lanes 9–10: *Shigella* primer 2.8 μL + *B. cereus* primer 2.4 μL; Lanes 11–12: *Shigella* primer 3.2 μL + *B. cereus* primer 2.4 μL. (**D**) RPA amplification results of optimal concentration combination in (**C**), Lane M: DNA marker DL2000; Lane 0: negative control; Lanes 1–2: *Shigella* primer 2 μL + *B. cereus* primer 1.6 μL.

Firstly, the same concentration of primers was set to make the electrophoretic bands of the amplification products of *Shigella* and *B. cereus* clear and uniform. The brightest electrophoretic bands were obtained when 2 and 4 µL of *Shigella* and *B. cereus* RPA primers were added, respectively (Figure 3A). However, the target bands were inconsistent after D-RPA amplification with this addition combination (Figure 3B). Therefore, we continued to adjust the primer concentration. In the first three groups in Figure 3C, the brightness of the two bands in lanes 3–4 tended to be the same when the concentration of *Shigella* RPA primer was controlled to be constant and that of the *B. cereus* RPA primer was gradually decreased. In the latter three groups, the difference in the brightness of the electrophoretic bands of the amplified products was larger when the concentration of the RPA primer of *B. cereus* was constant and that of *Shigella* was gradually increased. The optimal primer concentrations were finally determined to be 2 μL for *Shigella* and 1.6 μL for *B. cereus* to make the respective reactions as independent of each other as possible and to make the amplification results converge (Figure 3D).

**Figure 4:**
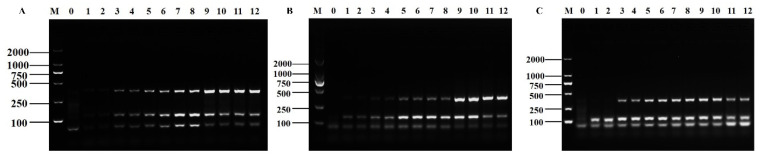
Optimization results of reaction time (**A**), reaction temperature (**B**), and Mg^2+^ concentration (**C**) of the D-RPA reaction system. Lane M: DNA marker DL2000; Lane 0: negative control; Lanes 1–2: 10 min; Lanes 3–4: 15 min; Lanes 5–6: 20 min; Lanes 7–8: 25 min; Lanes 9–10: 30 min; Lanes 11–12: 35 min. Lanes 1–2: 30 °C; Lanes 3–4: 33 °C; Lanes 5–6: 36 °C; Lanes 7–8: 39 °C; Lanes 9–10: 42 °C; Lanes 11–12: 45 °C. Lanes 1–2: 1 μL; Lanes 3–4: 1.5 μL; Lanes 5–6: 2 μL; Lanes 7–8: 2.5 μL; Lanes 9–10: 3 μL; Lanes 11–12: 3.5 μL.

The reaction times of 10, 15, 20, 25, 30, and 35 min were evaluated. With the increase in the reaction time, the electrophoretic bands of the *Shigella* amplification products gradually brightened and then darkened and those of the *B. cereus* amplification products gradually brightened (Figure 4A). According to the peak area values (Supplementary Material Table S2), the optimum reaction time was determined to be 25 min. The D-RPA assay was conducted under isothermal conditions between 30–45 °C. As the reaction temperature was increased, the electrophoretic bands of the *Shigella* amplification products brightened and then darkened, and those of the *B. cereus* amplification products continuously brightened (Figure 4B). According to the peak area values (Supplementary Material Table S3), the optimal reaction temperature condition was 42 °C. Subsequently, six different MgOAc additions of 1, 1.5, 2, 2.5, 3, and 3.5 μL were tested. The electrophoretic bands of *Shigella* and *B. cereus* RPA amplification products showed a weak trend of gradually brightening and then darkening as the Mg^2+^ concentration was increased (Figure 4C). According to the peak area value (Supplementary Material Table S4) and the principle of cost saving, the optimal MgOAc addition was selected as 2.5 μL.

### 3.4. Specificity and Sensitivity of the Method

**Figure 5:**
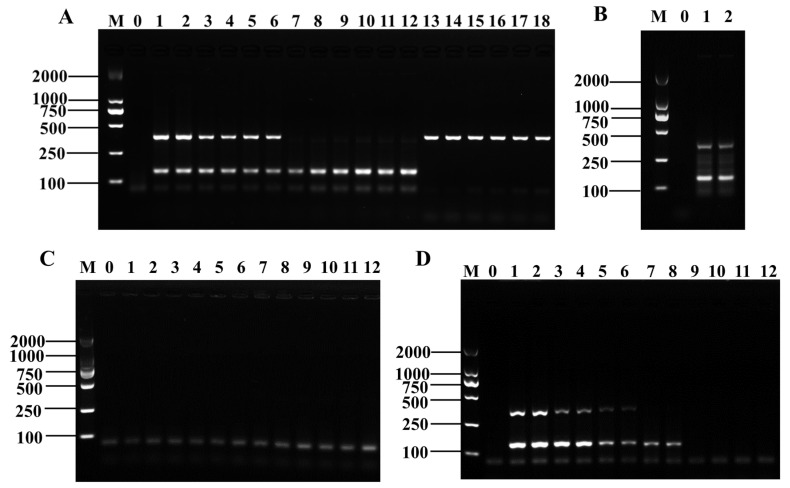
The specific verification results of the D-RPA reaction system. (**A**) Results of specificity verification of three *Shigella* and three *B. cereus* strains, Lane M: DNA marker DL2000; Lane 0: negative control; Lanes 1–2: *Shigella* (CMCC 51105) + *B. cereus* (CMCC 63303); Lanes 3–4: *Shigella* (72) + *B. cereus* (121); Lanes 5–6: *Shigella* (92) + *B. cereus* (124); Lanes 7–8: *Shigella* (CMCC 51105); Lanes 9–10: *Shigella* (72); Lanes 11–12: *Shigella* (92); Lanes 13–14: *B. cereus* (CMCC 63303); Lanes 15–16: *B. cereus* (121); Lanes 17–18: *B. cereus* (124). (**B**) Specific verification results using mixed DNA as a template, Lane M: DNA marker DL2000; Lane 0: negative control; Lanes 1–2: Mixed DNA of eight foodborne pathogenic bacteria. (**C**) Specific verification results using a single DNA as a template, Lane M: DNA marker DL2000; Lane 0: negative control; Lanes 1–2: *E. coli* O157:H7; Lanes 3–4: *V. parahaemolyticus*; Lanes 5–6: *Salmonella*; Lanes 7–8: *E. sakazakii*; Lanes 9–10: *L. monocytogenes*; Lanes 11–12: *S. aureus*. (**D**) The sensitivity verification results of the D-RPA reaction system. Lane M: DNA marker DL2000; Lane 0: negative control; Lanes 1–2: 30 ng/μL; Lanes 3–4: 3 ng/μL; Lanes 5–6: 0.3 ng/μL; Lanes 7–8: 0.03 ng/μL; Lanes 9–10: 0.003 ng/μL; Lanes 11–12: 0.0003 ng/μL.

Genomic DNA from 12 pathogenic bacteria suspensions (10^7^ CFU/mL) was extracted as a template for the assay to assess the specificity of D-RPA amplification. The results showed that three strains of *Shigella* and three strains of *B. cereus* could be successfully detected, and no cross-reaction occurred (Figure 5A). While using only mixed DNA as a template, the *Shigella* and *B. cereus* D-RPA systems were able to successfully amplify the target product (Figure 5B,C). This result indicated that the D-RPA detection system is specific and resistant to interference.

DNA diluted to six different concentrations were used as the templates to determine the sensitivity of the D-RPA amplification method. The results showed that the electrophoretic bands of the RPA amplification products gradually darkened as the concentrations of *Shigella* and *B. cereus* DNA were decreased. When the concentration of *Shigella* genomic DNA was 0.03 ng/μL, no bands were produced in the electrophoresis gels, indicating that no amplification product of *Shigella* DNA was obtained. When the concentration of *B. cereus* DNA was 0.3 ng/μL, no bands were produced in the electrophoresis gels, indicating the absence of the amplification product of *B. cereus* DNA (Figure 5D and Supplementary Materials Figure S1). The peak area values were consistent with the electrophoretic band brightness (Supplementary Materials Table S5). Therefore, the detection limits of the D-RPA reaction system were 30 and 300 pg for *Shigella* and *B. cereus*, respectively.

### 3.5. Application of D-RPA in Actual Sample Detection

**Figure 6:**
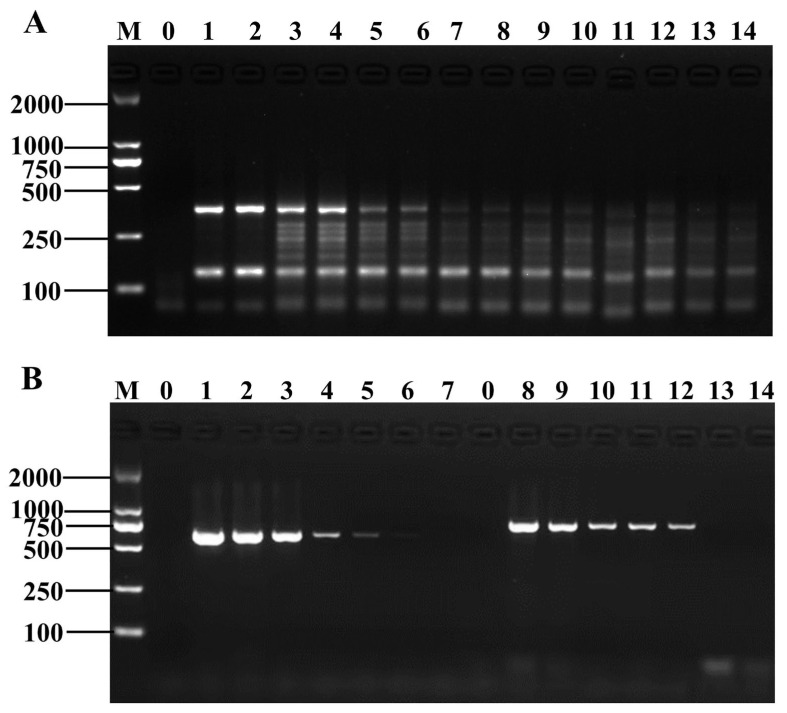
Actual sample verification. (**A**) Amplification results of D-RPA reaction, Lane M: DNA marker DL2000; Lane 0: negative control; Lanes 1–2: 10^7^ CFU/mL; Lanes 3–4: 10^6^ CFU/mL; Lanes 5–6: 10^5^ CFU/mL; Lanes 7–8: 10^4^ CFU/mL; Lanes 9–10: 10^3^ CFU/mL; Lanes 11–12: 10^2^ CFU/mL; Lanes 13–14: 10^1^ CFU/mL; (**B**) Amplification results of the conventional PCR reaction, Lane M: DNA marker DL2000; Lane 0: negative control; Lane 1–7: *Shigella* (10^7^–10^1^ CFU/mL); Lanes 8–14: *B. cereus* (10^7^–10^1^ CFU/mL).

DNA was extracted from artificially contaminated frozen green beans and then subjected to conventional PCR and D-RPA amplification separately to ensure the applicability and accuracy of the D-RPA reaction system in actual sample detection. The D-RPA system was also validated for the presence of *Shigella* and *B. cereus* with detection limits of 2.7 × 10^1^ and 5.2 × 10^2^ CFU/mL, respectively (Figure 6A and Supplementary Materials Figure S2), which were slightly better than the conventional PCR assay (Figure 6B), indicating that the D-RPA system is reliable for the detection of *Shigella* and *B. cereus* and can be applied to the detection of *Shigella* and *B. cereus* in actual food samples.

## 4. Discussion

*Shigella* and *B. cereus* are two common foodborne pathogenic bacteria, and the establishment of an accurate and efficient detection method is of great importance for the prevention of foodborne diseases. The RPA technique has attracted the attention of researchers because of its unique advantages, such as rapidity and reactivity at room temperature. Although studies on the detection of common foodborne pathogens using the single RPA technique have been reported, these studies are few in number and lack sufficient depth, and even fewer articles on *Shigella* and *B. cereus* studies have been published. Zheng et al. [31] established RPA-LFD for the detection of *Shigella* and enteroinvasive *Escherichia coli* with detection limits of 1.46 × 10^3^ CFU/mL and 1.63 × 10^3^ CFU/mL in cucumber-contaminated samples, respectively. Liu et al. [32] established a real-time single-weight RPA system for the rapid detection of *B. cereus* based on the 16S RNA gene, which had a detection limit of 1.5 × 10^4^ CFU/g in artificially contaminated rice. The D-RPA system for *Shigella* and *B. cereus* established in this study can detect both pathogenic bacteria at similar temperatures and time with slightly better detection limits.

The exploration of multiplex RPA technology in the detection of foodborne pathogenic bacteria is relatively limited given the many difficulties that remain unaddressed. First of all, because the design principles of RPA primers are different from those of conventional PCR, no special RPA primer design software is available thus far, and multiple pairs of primers have to be designed for multiple RPA reactions. The increased binding possibilities between each primer must be considered, and avoiding the formation of structures, such as primer dimers, is difficult. Considering that primer design is a prerequisite for successful RPA reactions and a key to success, the preliminary primer design requires extensive synthetic screening and experimental verification, which may be highly expensive and time-consuming [33]. The design of specialized software for RPA primers will be a direction that needs to be investigated and refined such that RPA reactions can then be used with increased efficiency in various assays. Moreover, the addition of multiple pairs of specific primers into the multiplex RPA system increases the complexity of the reaction system, and the amplification efficiency of different primers is different. Although the reaction system can be optimized by optimizing each condition to achieve a desirable effect, making the reaction efficiency of each pair of primers in the same reaction system consistent appears to be difficult.

The detection of multiplex RPA amplification products also introduces new technical difficulties. The commonly used AGE technique, which must be opened at the end of product amplification, can easily lead to aerosol contamination. Furthermore, the AGE technique takes approximately half an hour to yield results. This situation undoubtedly affects the efficiency of detection and makes achieving rapid detection difficult. Lateral flow test strip technology is considered to be a rapid and portable means, but its demand for high sensitivity is still difficult to meet. Converting assays into quantitative data by using commercial readers is possible, but the high cost and difficult portability of readers limit their availability and use [34], and technical difficulties are encountered in combining multiplex RPA technology with visualization techniques. Combining multiplex RPA technology with other product analysis technologies and breaking through the technical bottleneck will be the direction we need to continuously work on. However, we believe that with the development of biosensors and rapid field detection technologies, additional new portable high-throughput technologies and methods for field rapid detection will be applied for the detection of foodborne pathogenic bacteria [35].

In conclusion, multiple pathogenic bacteria often co-exist in food, and multiplex detection technology can not only improve detection efficiency and save detection costs but also increase the accuracy of detection results. Therefore, this technology has a good development prospect. However, the current implementation of multiplex RPA technology faces many challenges, which will be the focus of future research. In this study, we established a D-RPA detection system for *Shigella* and *B. cereus.* This method is an exploration of the use of multiplex RPA technology and high-throughput detection technology for the detection of foodborne pathogens. However, the application of this technology is still in the initial stage, and many areas are not covered or not deeply investigated.

## 5. Conclusions

In this study, we successfully designed and screened the specific RPA primer *ipaH7*-3F/R for *Shigella* and *nheB*-3F/R for *B. cereus*, and established the first D-RPA detection system for *Shigella* and *B. cereus* by using RPA technology combined with electrophoresis. The system can effectively amplify at 42 °C for 25 min and complete all detection within 1 h. It has good sensitivity and specificity and has been successfully applied in the detection of actual samples. The use of electrophoresis for RPA amplification reduces the cost of detection, and given the complexity of the D-RPA reaction system, avoiding probe design helps simplify the reaction system and reduce the possibility of false positives.

## Figures and Tables

**Table 1:** Information of bacterial strains used for specificity tests.

Species	ID of Strains
*Shigella*	CMCC[B] 51105
	72 *
	92 *
*Bacillus cereus*	CMCC[B] 63303
	121 *
	124 *
*Escherichia coli* O157:H7	NCTC 12900
*Salmonella*	CMCC[B] 50094
*Vibrio parahaemolyticus*	ATCC 17802
*Enterobacter sakazakii*	ATCC 29544
*Listeria monocytogenes*	CMCC[B] 54002
*Staphylococcus aureus*	CMCC[B] 26003

* The strains were isolated and preserved from food samples in our laboratory.

## Data Availability

All data generated in this study are included in this article and its supplementary materials.

## Supplementary Materials

The following supporting information can be downloaded at: https://www.mdpi.com/article/10.3390/foods12091889/s1, Table S1: All primer sequences. Table S2: Gray analysis of electrophoretic bands of reaction time optimization results of the D-RPA reaction system. Table S3: Gray analysis of electrophoretic bands of reaction temperature optimization results of the D-RPA reaction system. Table S4: Gray analysis of electrophoretic bands of Mg^2+^ concentration optimization results of the D-RPA reaction system. Table S5: Gray analysis of electrophoretic bands of sensitivity verification results of the D-RPA reaction system. Figure S1. Results of the sensitivity validation of the D-RPA reaction system with grayscale analysis of the electrophoretic bands. (A) *Bacillus cereus*; (B) *Shigella*. Figure S2. Results of the D-RPA reaction system in actual samples with grayscale analysis of the electrophoretic bands. (A) *Bacillus cereus*; (B) *Shigella*.
