# Supplementary material for: A New Duplex Recombinase Polymerase Amplification (D-RPA) Method for the Simultaneous and Rapid Detection of Shigella and Bacillus cereus in Food
Source: Foods. 2023 May 4;12(9):1889. doi: 10.3390/foods12091889 (PMC10178236; doi:10.3390/foods12091889)
Supplement: Supplementary file 1 [file foods-12-01889-s001.zip › foods-2297644-supplementary.pdf]

Supplementary Materials

Table S1. All primer sequences.

Primer Name			Sequence（5′→3′）	Product size (bp)	Reference
16S rDNA universal primer sequence		27-F	AGAGTTTGATCCTGGCTCAG	1516	[1]
		1429-R	TACGGCTACCTTGTTACGACTT		
RPA candidate primer sequence	*Shigella*	*ipaH*-1F	CCTTTTCGATAATGATACCGGCGCTCTGCT	213	This study
		ipaH-1R	GTCACTCCCGACACGCCATAGAAACGCATT		
		ipaH-2F	CCTTGGCGCTTTCCTCGCTACCTGTACTCC	237	
		ipaH-2R	CTGCGCTTAGTGATTTGATGGTGTCTGGTA		
		ipaH-3F	GGCAGCCTGGTTTCCTGAAGCAGATCGTCG	137	
		ipaH-3R	CGGAGGTATTGCGTGCAGAGACGGTATCGG		
	B. cereus	*nheA*-1F	AGCTTAACGAATCATCAAAAGTTCGCAAAGGC	252	
		nheA-1R	TAAATCTTGCTCCATACTCTCTTGGATGCTCTG		
		nheA-2F	CAGGGTTATTGGTTACAGCAGTATCTACGA	341	
		nheA-2R	GCTATTAAATCTAGTGCTGTATCTCATCAT		
		nheA-3F	GAATCATCAAAAGTTCGCAAAGGCGAATGTG	161	
		nheA-3R	GCTGATCTTCATTTACGTTTCCTGCTAGTTCA		
		nheB-1F	AATTCAGCACCAAGATACAGCTAGAGGAAATG	174	
		nheB-1R	CCTTTCGTAAGAGTTGCTTTATCCTTTGCATC		
		nheB-2F	AATGGCTCTATCAGCACTTATGGCAGTATTTG	312	
		nheB-2R	CCTCTAGCTGTATCTTGGTGCTGAATTACTTTC		
		nheB-3F	TAAAGCGCAAGTGGATCAGTTAGTAGAAGACT	361	
		nheB-3R	ATCAAGCTCTTTCTTCGCTAATACGATTCCAG		
Ordinary PCR primer sequence	*Shigella*	*ipaH*-F	GTTCCTTGACCGCCTTTCCGATACCGTC	629	[2]
		ipaH-R	GCCGGTCAGCCACCCTCTGAGAGTAC		
	B. cereus	*nheB*-F	CTATCAGCACTTATGGCAG	770	[3]
		nheB-R	ACTCCTAGCGGTGTTCC		

Note: F, forward primer; R, reverse primer.

Table S2. Gray analysis of electrophoretic bands of reaction time optimization results of the D-RPA reaction system.

Lanes	Peak area of *Shigella*	Peak area of *Bacillus cereus*
1	934.698	553.456
2	1017.113	549.213
3	4355.477	3071.698
4	4346.598	3168.941
5	6006.477	4607.113
6	7550.184	5377.941
7	10617.184	7623.941
8	11040.305	7851.82
9	9429.77	11998.355
10	7851.477	11691.355
11	7669.062	11388.062
12	7616.012	11522.82

Table S3. Gray analysis of electrophoretic bands of reaction temperature optimization results of the D-RPA reaction system.

Lanes	Peak area of *Shigella*	Peak area of *Bacillus cereus*
1	2862.062	488.799
2	2565.82	453.556
3	4364.355	1248.284
4	4963.941	1464.577
5	9000.305	3940.82
6	9163.77	4026.113
7	8791.355	4599.698
8	7527.648	3614.698
9	9222.062	10537.477
10	9648.477	11029.527
11	5670.234	10209.234
12	5132.82	9954.82

Table S4. Gray analysis of electrophoretic bands of Mg^{2+} concentration optimization results of the D-RPA reaction system.

Lanes	Peak area of *Shigella*	Peak area of *Bacillus cereus*
1	8687.548	363.385
2	9489.598	390.749
3	9664.355	5362.355
4	9933.184	5340.648
5	9398.062	6948.234
6	9720.062	7266.355
7	9060.941	7819.941
8	9889.598	8218.062
9	9896.184	8738.234
10	9665.062	8771.477
11	8824.891	6360.234
12	8423.225	6651.719

Table S5. Gray analysis of electrophoretic bands of sensitivity verification results of the D-RPA reaction system.

Lanes	Peak area of *Shigella*	Peak area of *Bacillus cereus*
1	15572.962	10196.962
2	16263.669	10784.134
3	14077.326	4341.426
4	13868.912	4125.012
5	6751.669	1862.648
6	4870.255	1318.698
7	5770.962	
8	5561.255	

A

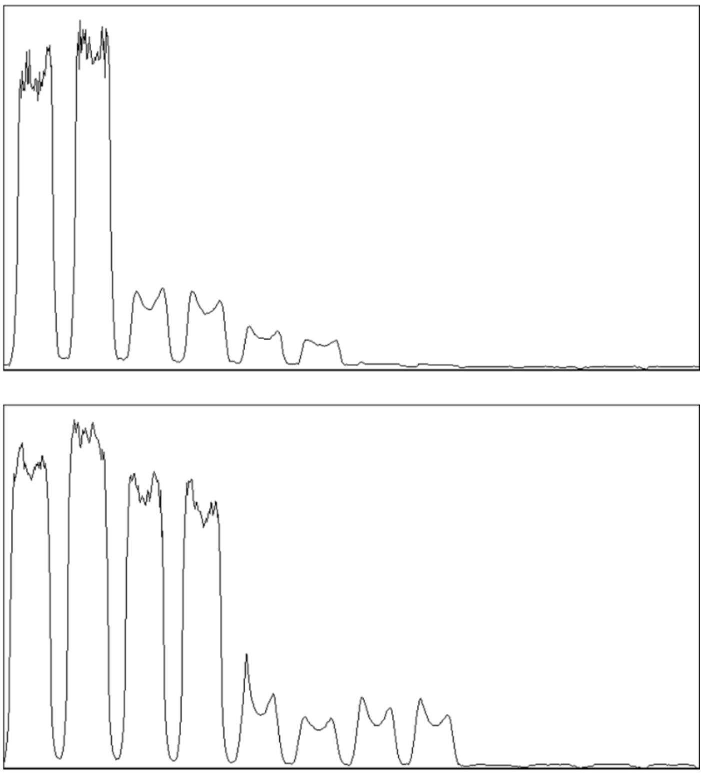

B

Figure S1. Results of the sensitivity validation of the D-RPA reaction system with grayscale analysis of the electrophoretic bands. (A) *Bacillus cereus*; (B) *Shigella.*

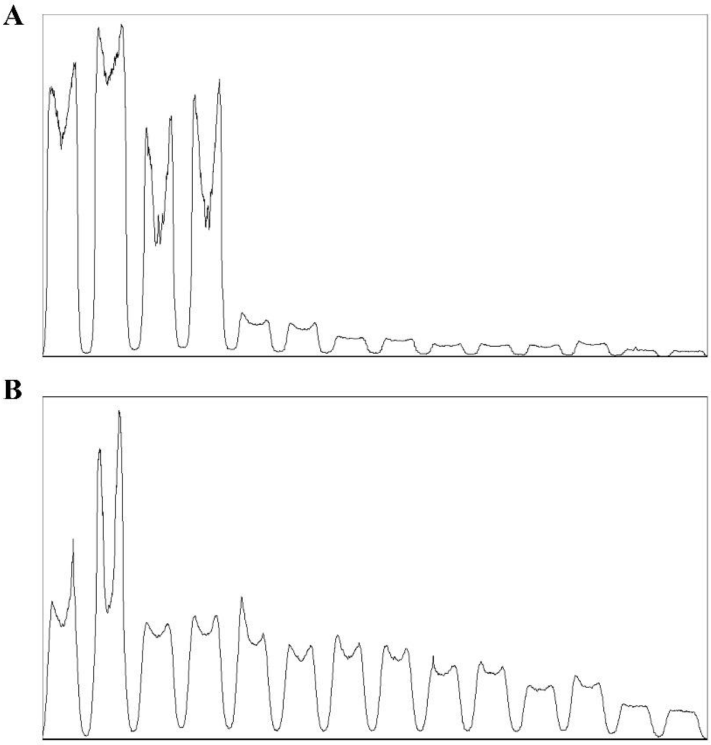

Figure S2. Results of the D-RPA reaction system in actual samples with grayscale analysis of the electrophoretic bands. (A) *Bacillus cereus*; (B) *Shigella.*

Reference

1. Frank, J.A.; Reich, C.I.; Sharma, S.; Weisbaum, J.S.; Wilson, B.A.; Olsen, G.J. Critical evaluation of two primers commonly used for amplification of bacterial 16S rRNA genes. *Appl. Environ. Microbiol.* **2008**, *74*, 2461-2470.
2. General Administration of Quality Supervision, Inspection and Quarantine of the People's Republic of China. Rapid detection of many pathogenic bacteria in food PCR method. **2007**, *SN/T 1869-2007*, 15P.;A14.
3. Hansen, B.M.; Hendriksen, N.B. Detection of enterotoxic *Bacillus cereus* and *Bacillus thuringiensis* strains by PCR analysis. *Appl. Environ. Microbiol.* **2001**, *67*, 185-189.
